# Scission, Cross-Linking, and Physical Relaxation during Thermal Degradation of Elastomers

**DOI:** 10.3390/polym11081280

**Published:** 2019-07-31

**Authors:** Maha Zaghdoudi, Anja Kömmling, Matthias Jaunich, Dietmar Wolff

**Affiliations:** Bundesanstalt für Materialforschung und -prüfung (BAM), 12200 Berlin, Germany

**Keywords:** ageing, scission, cross-linking, compression set, physical relaxation

## Abstract

Elastomers are susceptible to chemical ageing, i.e., scission and cross-linking, at high temperatures. This thermally driven ageing process affects their mechanical properties and leads to limited operating time. Continuous and intermittent stress relaxation measurements were conducted on ethylene propylene diene rubber (EPDM) and hydrogenated nitrile butadiene rubber (HNBR) samples for different ageing times and an ageing temperature of 125 °C. The contributions of chain scission and cross-linking were analysed for both materials at different ageing states, elucidating the respective ageing mechanisms. Furthermore, compression set experiments were performed under various test conditions. Adopting the two-network model, compression set values were calculated and compared to the measured data. The additional effect of physical processes to scission and cross-linking during a long-term thermal exposure is quantified through the compression set analysis. The characteristic times relative to the degradation processes are also determined.

## 1. Introduction

Elastomers are widely used in industry and are particularly often applied in sealing due to their ability to undergo high elastic deformation. Moreover, elastomeric materials are easy to use. In operation, these materials are exposed to various thermo-mechanical and chemical factors, which affect their resilience and their sealing ability. Thus, an understanding of the long-term thermal and thermo-mechanical degradation of elastomeric components is of major interest to predict their lifetime in operating conditions. Degradation due to ageing is caused by both physical and chemical processes [1]. Physical ageing describes the observed changes in properties of glassy materials as a function of storage time, at a temperature below the glass transition (T_g_) in a stress-free configuration [2]. It is well known that these materials are in a non-equilibrium state below T_g_ and that any thermodynamic system brought out of equilibrium spontaneously evolves to re-establish its equilibrium state. Physical ageing is a reversible process associated with macromolecular rearrangement. This occurs when the chains in the perturbed state attempt to reach a new equilibrium configuration through the movement of entanglements and the relaxation of dangling ends [2,3]. A common measure of physical ageing relies on monitoring the recovery of a thermodynamic quantity such as volume or enthalpy [4]. 

For rubbers which are typically used above T_g_ but still show long relaxation times, physical effects are relevant for the mechanical performance. Unlike physical ageing, chemical ageing is an irreversible process leading to oxidation, chain scission, and the formation of new cross-links [1]. Usually, both chain scission and cross-linking occur during ageing of a specific rubber material, but typically one process dominates and leads to characteristic changes of the material properties. In general, during ageing of ethylene propylene diene rubber (EPDM), both chain scissions (mainly in the propylene segments [5]) and cross-linking reactions (mostly via the termonomer [6]) take place [7]. In hydrogenated nitrile butadiene rubber (HNBR), cross-linking reactions dominate [7,8]. Chain scission reactions lower the cross-link density and, thus, result in softening and loss of elastic properties, while cross-linking during ageing leads to stiffness increase and embrittlement of the material [1]. Most conventional polymer analysis methods yield averaged results over these two processes. For example, the hardness increases if the cross-link density increases, and decreases when chains are broken [9]. That means that if a slight hardness increase is measured, it is not clear whether it is only caused by a slightly increased cross-link density, or if both chain scission and cross-linking are occurring, but the influence of cross-linking slightly outweighs that of chain scissions. To correctly assess the amount of degradation in a sample, e.g., for lifetime predictions, it is therefore important to analyse the contribution of chain scissions and cross-linking in detail. Direct (analytical) methods such as infrared spectroscopy, nuclear magnetic resonance, Raman spectroscopy, and chemiluminescence as well as indirect (mechanical) methods such as tensile tests and hardness can evaluate the material changes during ageing [10,11,12,13]. The main purpose of the understanding of the molecular processes and the complex interactions that occur during ageing with these methods is to predict the lifetime under service condition. Note that the analysis of the detailed chemical changes through the abovementioned analytical methods is beyond the scope of the present investigation. The focus in the present study relies on methods that are especially suitable for the analysis of sealing components and their degradative processes, namely the compression set (CS) measurement and the combination of intermittent and continuous stress relaxation [14]. CS is a measure for resilience and is determined from the recovered height after ageing in compression. The detailed procedure is described, e.g., in the standards DIN ISO 815 [15] and ASTM D395. Unlike many other analysis methods, CS is influenced additively by chain scission and cross-linking, as both effects lead to an increase of the measured CS: Broken chains lose their recovery potential, and new cross-links form in equilibrium with the compressed geometry, thus fixing it. During continuous stress relaxation measurements (cf. DIN ISO 3384 [16]), the formation of new cross-links has practically no influence on the measured force since the resulting new chains are formed in a relaxed state [14], but chain scissions lead to a force reduction, as the entropically stored force in the cleaved chain is dissipated by relaxation of the two chain ends. On the other hand, the force measured with intermittent stress relaxation measurements is influenced by both chain scission and cross-linking. By combining both relaxation measurements, it is thus possible to identify the contribution of each ageing effect. When evaluating data from these measurements, care has to be taken to avoid diffusion limited oxidation (DLO) effects [17,18,19,20,21]. These effects may occur during accelerated ageing when the oxygen consumption in the sample is faster than oxygen diffusion into the sample. DLO effects lead to heterogeneous samples with a less-aged interior and thus to problems when attempting to predict the lifetime of components under service conditions. Extrapolations for lifetime predictions are mainly based on the time–temperature shift (TTS) [22] or/and the Arrhenius approach [23] as mentioned in the ISO 11346 standard [24] for the estimation of rubber lifetime. Recently, lifetime predictions were performed with the combination of mathematical algorithms and short-time mechanical tests [25,26]. However, the presence of DLO effects slows the ageing rate and can distort the data for the lifetime prediction analysis. Therefore, the present study has been performed on DLO-free samples as assessed in our previous publications [21,27]. Another encountered challenge during data analysis is to separate physical and chemical relaxation processes. To separate physical relaxation, experiments found in literature were performed either in nitrogen or first at low temperature or short time where the chemical relaxation is neglected and then the resulting relaxation is subtracted at high temperature or longer time via the time–temperature superposition technique [28,29,30]. In this paper, the contributions of chain scission and cross-linking at different ageing states for EPDM and HNBR are analysed and a new approach to separate physical and chemical processes that occur during stress relaxation is proposed through the analysis of compression set. Characteristic times relative to the different degradation states are determined. In the forthcoming research, this should simplify the identification of material parameters that are required to model the mechanical behaviour of elastomers during thermo-oxidative ageing.

## 2. Materials and Methods 

### 2.1. Materials

The commercial EPDM and HNBR rubbers were peroxide cured. The EPDM used contained 48 wt % ethylene, 4.1 wt % ethylidene norbornene (ENB), 90 phr carbon black fillers, and no plasticizer. The HNBR base polymer contained 36 wt % acrylonitrile and had an iodine number of 11. The HNBR was reinforced with 80 phr of filler (75 phr carbon black and 5 phr silica) and softened with 5 phr of plasticizer. Due to confidentiality constraint, the detailed compound ingredients are not provided here. O-rings and segments with a cord diameter of 10 mm and an inner diameter of 190 mm were investigated, as well as sheets with a thickness of 2 mm. All samples were aged in air circulating ovens at 125 °C for ageing times of 2 h, 6 h, 1 day, 2 days, 3 days, 10 days, and 31 days. For EPDM samples, two additional ageing times of 100 days and 185 days were required to obtain more strongly aged samples. For measuring compression set, two half O-rings of each material were aged compressed by 25% between stainless steel plates. The uncompressed O-ring segments with a length of 10 mm and the sheets with a thickness of 2 mm were aged in a stress-free state. 

### 2.2. Methods

#### 2.2.1. Continuous Stress Relaxation

Continuous stress relaxation measurements were conducted at 125 °C on O-ring segments with a length of approx. 40 mm that were first conditioned thermally and mechanically according to the standard DIN ISO 3384 [16] and then aged compressed by 25%. Three O-ring segments per material and temperature were tested using the stress relaxation rigs EB 02 in a cell oven EB 22 from Elastocon. Isothermal force measurements (Brämhult, Sweden) were performed every 10 seconds for the first hour, every minute for the next 12 h, and afterwards every 10 minutes (without changing the load). 

#### 2.2.2. Intermittent Stress Relaxation

**Figure 1:**
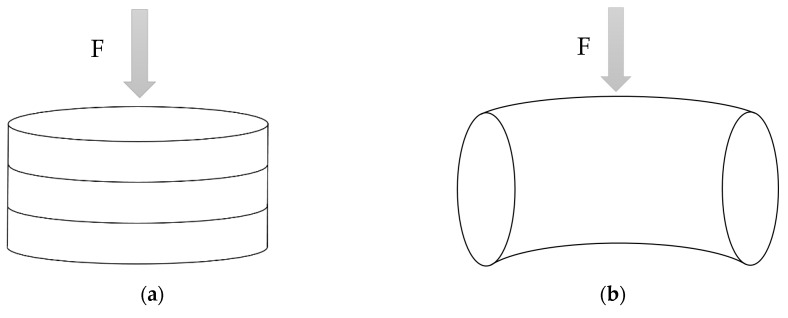
Schematic representation: (**a**) Stacked disks with axial loading; (**b**) O-ring segment with radial loading.

Intermittent stress values were measured on both O-ring segments with a length of 10 mm and disks with 10 mm diameter that were die-cut from 2 mm thick sheets and stacked to give a height of 6 mm (see Figure 1). After stress free ageing, the O-ring segments or disk stacks were rapidly compressed by 25% and the necessary force was measured by means of a GABO Eplexor 500 device (Gabo, Ahlden, Germany) with a 500 N load cell. Silicon oil was used as a lubricant between the top and bottom of the disk stack. The relaxation test was continued for one hour. To ensure the reliability of the data obtained, each test was performed three times using three different samples. For each intermittent stress relaxation measurement, a new sample was taken. This ensured that the collected data were not influenced by previous compression (Mullins effect [31]) or through the difference between the relaxation time of the sample and the test time.

#### 2.2.3. Compression Set

Compression set provides information about the recovery behaviour of samples. It is calculated by means of Equation (1):

(1) $$CS\left(\%\right)=\frac{h_{0}-h_{2}}{h_{0}-h_{1}}\cdot 100\%\;,$$

where $h_{0}$, $h_{1},$ and $h_{2}$ are respectively the initial, the compressed, and the recovered height of the sample. The recovered height, $h_{2},$ is strongly time dependent and is measured after three different times. According to standards ASTM D395 and DIN ISO 815-1 [15], the recovered height, $h_{2},$ is first measured after 30 min. However, after 30 min the recovery is still quite fast, and the measurement is influenced by the exact measurement time and the time necessary for disassembling the plates. Therefore, in our previous studies [21,32], CS was measured after five days, when the measured values are much closer to equilibrium. However, it has been shown that the recovery proceeds further, and, therefore, the accelerated recovery as proposed by Gillen et al. [33] was adopted, i.e., the samples were placed in an oven for one day at 100 °C to accelerate the recovery towards equilibrium. This ensures that the measured CS reflects only the irreversible chemical reactions (chain scission and cross-linking). The recovered height of O-ring segments was measured at 7–10 positions using a calliper and CS was calculated from the average value. The maximum error of CS measurements was about ±3%.

## 3. Results and Discussion 

### 3.1. Geometry Comparison 

The intermittent stress relaxation measurements were performed on a GABO Eplexor device with 500 N static force. As the used O-rings have a line force of 300 N/cm for 25% compression and harden during ageing, only O-ring segments with a length of 10 mm could be tested. However, it is very difficult to prepare segments with exactly 10 mm length, as the rubber bends during cutting. Therefore, disks with a diameter of 10 mm were die-cut from sheet material and three disks were stacked to obtain a volume similar to the O-ring segment. This sample preparation yielded a very uniform geometry. Using the sheet material had the additional advantage of excluding the slight DLO effects that were detected previously in HNBR O-rings aged at 125 °C [21,27]. In order to determine possible deviations between the two sample configurations, comparative tests were made. Table 1 shows the relative deviation of the normalized force after compression between disk stack and O-ring segment at relevant ageing times and is calculated using Equation (2):

(2) $$\mathrm{Relative}\;\mathrm{deviation}\;\left(\%\right)=\left({\left[\frac{F_{i}}{F_{0}}\right]}_{a}-{\left[\frac{F_{i}}{F_{0}}\right]}_{r}\right)\cdot \frac{100}{{\left[\frac{F_{i}}{F_{0}}\right]}_{a}},$$

where subscripts *i*, 0, *a*, and *r* denote intermittent, quantity at time t=0, axial, and radial, respectively (cf. Figure 1).

Slight deviations of up to 5% are observed, possibly due to lower friction between the disks compared to the coherent volume of the O-ring segment.

### 3.2. Continuous and Intermittent Stress Relaxation 

**Figure 2:**
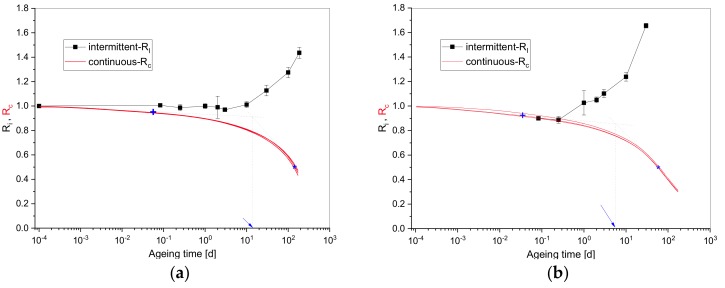
Continuous and intermittent normalized stress relaxation data at 125 °C for: (**a**) EPDM; (**b**) HNBR.

The continuous and the intermittent normalized compression stress measurements for EPDM and HNBR at 125 °C are shown in Figure 2 as a function of ageing time. The normalized continuous stress, $R_{c},$ is the ratio of the continuously measured force, $F_{c},$ to the initial force, $F_{0},$ at t=0, starting when the full compression was achieved. Likewise, the normalized intermittent stress, $R_{i},$ is the ratio of the measured force, $F_{i},$ to the initial force, $F_{0},$ of the unaged sample.

As outlined in the introduction, during continuous stress relaxation measurements, the formation of new cross-links has practically no influence on the measured force, but chain scissions lead to a force reduction. On the other hand, in intermittent stress relaxation measurements the normalized stress, $R_{i},$ is a result of the net effect of cross-linking and scission reactions.

In both graphs of Figure 2, the continuous force, $R_{c},$ decreases with ageing time due to chain scissions, while the intermittent force, $R_{i},$ increases for longer ageing times, indicating an excess of cross-linking reactions. Furthermore, the continuous relaxation exhibits two regions [34], a flat decrease at the beginning and a stronger decrease at higher ageing times. According to Johlitz et al. [35,36], the first phase correlates with physical relaxation, i.e., chain rearrangements under the compression load. For the investigated materials, the duration of dominant physical relaxation is marked by the cross (+) in Figure 2. The cross presents the end of the linear region and is defined as the time at which the measurement data deviate by 1% from the straight line. The second phase with more pronounced force decrease is associated with chain scission reactions that lower the number of stress-bearing chains in the sample cross-section. The onset or induction time of the dominant chemical reactions can be estimated from the intersection of the two linear regions, as indicated by the dotted lines and the arrow in Figure 2. The line for the first section was obtained by fitting a tangent to the linear region starting from a fixed point which corresponds to the first recorded value of $R_{c}$ with a slope of −3.9 × 10^−3^ and −2.4 × 10^−2^, respectively for EPDM and HNBR, while the second line was obtained by fitting a tangent to the half-time relaxation at 50% force loss (marked by a star (*) in Figure 2) with a slope of −3.3 × 10^−3^ and −6.2 × 10^−3^, respectively for EPDM and HNBR. From the slope values of the two linear regions of HNBR compared to EPDM, a shorter physical relaxation duration and a faster degradation process is to be expected. 

Physical relaxation durations and chemical induction times as well as relaxation half-times are summarized in Table 2. The obtained times are lower for HNBR, which degrades faster than EPDM [7,21].

When taking a closer look at the intermittent force curve of EPDM, two regions can be identified as well. In the beginning, the intermittent force decreases slightly up to 3 d, indicating dominant chain scission reactions. For longer ageing times, the intermittent force increases due to dominant cross-linking. On the other hand, the intermittent measurements of HNBR coincide with the continuous ones up to 0.25 d then increase rapidly due to dominant cross-linking.

### 3.3. Scission and Cross-Linking 

In order to investigate the contributions of chain scission and cross-linking in more detail, an approach outlined by [14] was adopted, in which the combined continuous and intermittent stress relaxation measurements were used to separate the effects of chain scission and cross-linking. The governing equations from the statistical theory of rubber elasticity are as follows [6].

(3) $$F_{c}=A\cdot n\left(t\right),$$

(4) $$F_{0}=A\cdot n\left(0\right),$$

(5) $$R_{c}=\frac{F_{c}}{F_{0}}=\frac{n\left(t\right)}{n\left(0\right)},$$

(6) $$F_{i}=A\cdot \left[n\left(t\right)+n^{cro}\left(t\right)\right],$$

(7) $$R_{i}=\frac{F_{i}}{F_{0}}=\frac{n\left(t\right)+n^{cro}\left(t\right)}{n\left(0\right)}.$$

Subscripts, *c*, *i*, *0*, and superscript, *cro*, denote continuous, intermittent, quantity at time t=0, and cross-link, respectively. $n\left(t\right)$ is the number of the remaining initial chains and $n^{cro}\left(t\right)$ is the number of newly formed cross-links. A is a constant with $A=\frac{k\cdot T}{L_{0}}\left(\lambda -\frac{1}{\lambda^{2}}\right)$. k is Boltzmann’s constant, T is the absolute temperature, $L_{0}$ is the initial length of the specimen, and λ is the stretch ratio ($\lambda =\frac{L}{L_{0}}$, L is the elongated length). 

**Figure 3:**
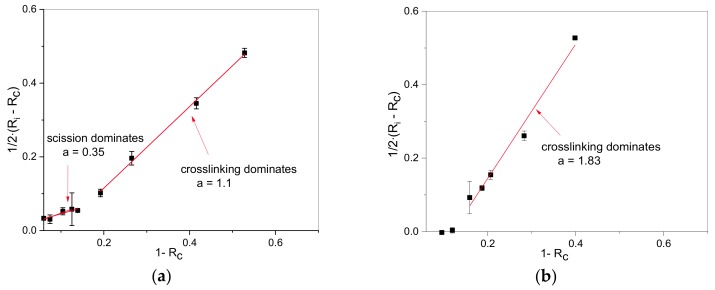
Evolution of cross-linking versus scission at 125 °C for: (**a**) EPDM; (**b**) HNBR.

The term $\left(1-R_{c}\right)$ correlates to the number of chain scissions, and the difference $\frac{1}{2}\cdot (R_{i}-R_{c})$ correlates to additional cross-links. The term $\left(R_{i}-R_{c}\right)$ is divided by two, as the formation of one new cross-link includes two chains but should be considered as one event. When $\frac{1}{2}\cdot \left(R_{i}-R_{c}\right)$ is plotted over $\left(1-R_{c}\right)$ for different ageing states, a slope, *a*, larger or smaller than 0.5 indicates which process dominates, with a slope smaller than 0.5 indicating dominant chain scission reactions, and a slope larger than 0.5 indicating dominant cross-linking reactions. These plots are depicted in Figure 3. The graph of aged EPDM samples (Figure 3a) shows two linear regions, with a smaller slope indicating chain scission at the beginning, and a larger slope with dominant cross-linking for longer ageing times whereas cross-linking reactions dominate for HNBR (Figure 3b). This confirms the studies made on HNBR during thermal ageing [7,8]. For EPDM, the linear relationship at a shorter ageing time is in accordance with the findings of Landi et al. [6] for short-term ageing of EPDM with ENB terpolymer. 

**Figure 4:**
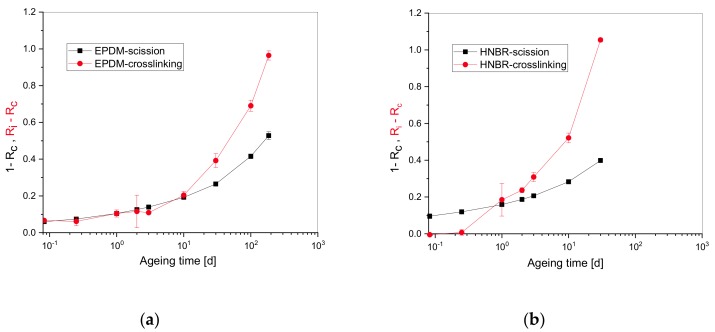
Evolution of cross-linking and chain scission versus ageing time for (**a**) EPDM and (**b**) HNBR.

Figure 4 shows the evolution of the amount of chain scission and cross-linking vs. ageing time, which is faster for HNBR. This explains the lower recorded relaxation half-time (cf. Table 2). For EPDM, chain scission and cross-linking are occurring with approximately equal impact at shorter ageing times. For HNBR, there is a short phase in the beginning with dominant scission. Later in the degradation process, cross-linking dominates especially for HNBR, while the influence of chain scission reactions competes with that of cross-linking for EPDM at longer ageing times. This means that during the ageing of EPDM, polymer chains are broken, and new network bridges are simultaneously formed.

Figure 4 shows that cross-linking exceeds scission after 10 and 1 d, respectively, for EPDM and HNBR. On the other hand, the graphically determined induction times (Figure 2 and Table 2) are 10 and 6 d for EPDM and HNBR, respectively, which correspond to scission values of 0.2 and 0.25 from Figure 4a,b. It should be kept in mind that the evolution of chain scission and cross-linking in Figure 4 are calculated with the two networks theory [14] while the graphically determined induction times (Figure 2 and Table 2) are only related to scission. 

### 3.4. Viscoelastic Contribution

Since chain scission is closely connected to viscosity, it is of interest to investigate the viscoelastic contribution to the force measured with intermittent stress relaxation. $F_{eq}$ is the force value at the end of each relaxation test period (after one hour) and correlates to the cross-link density, while $F_{visco}$ is the difference between the force at the beginning and at the end of each test and increases with the number of chain scissions [32]. The normalized $R_{eq}$ and $R_{visco}$ are the ratios of $F_{eq}$ and $F_{visco}$ to $F_{0}$ with respect to the unaged values and are defined by Equations (8) and (9):

(8) $$R_{eq}=\frac{{\left[\frac{F_{eq}}{F_{0}}\right]}_{aged}}{{\left[\frac{F_{eq}}{F_{0}}\right]}_{unaged}},$$

(9) $$R_{visco}=\frac{{\left[\frac{F_{visco}}{F_{0}}\right]}_{aged}}{{\left[\frac{F_{visco}}{F_{0}}\right]}_{unaged}}.$$

**Figure 5:**
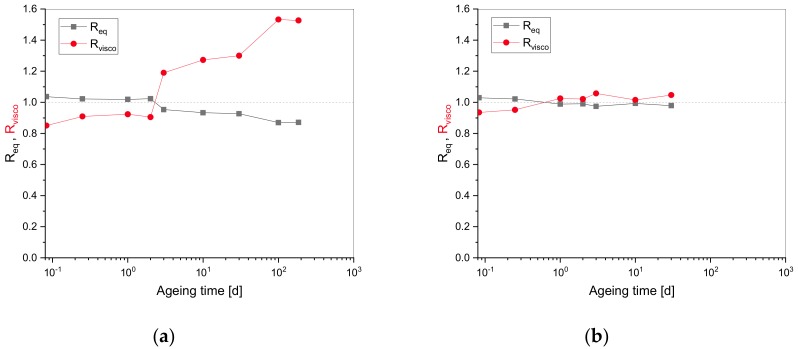
Influence of the viscoelastic contribution for (**a**) EPDM and (**b**) HNBR.

Figure 5 shows the evolution of $R_{eq}$ and $R_{visco}$ for EPDM and HNBR vs. ageing time.

A plateau was recorded for $R_{eq}$ at shorter ageing times. Afterwards, lower values were obtained with increased ageing times for EPDM. $R_{eq}$ correlates with the cross-link density. As at shorter ageing times chain scission reactions balance cross-linking, macroscopically, the system behaves like an unaged one (with the same cross-link density). On the other hand, when cross-linking is preponderant at longer ageing times, a higher cross-link density and, thus, an increase of $R_{eq}$ is expected. However, the chain scission effects seem to dominate, as $R_{visco},$ which correlates with chain scission, increases gradually up to ~1.77 times the initial value for EPDM at longer ageing times. In Figure 5b, $R_{visco}$ evolves quite the same as $R_{eq}$ for HNBR. Similar to EPDM, the effect of chain scissions seems to be stronger than expected from Figure 4, so that even the smaller contribution of chain scissions in HNBR could balance the effect of cross-linking. A possible explanation is that the initial compressive force $R_{i}$ is sensitive to cross-linking (Figure 2), whereas the short-time relaxation experiment (Figure 5) that reveals the viscoelastic properties is more sensitive to chain scission. Another possible reason for the different tendencies in Figure 4 and Figure 5 is that the increase of $R_{i}$ could also be due to the higher polarity and thus higher molecular attraction between chains [37]. For example, polymer oxidation through chain scission often leads to the formation of carbonyls that exhibit a much higher polarity than hydrocarbons. Thus, the higher polarity induced by oxidative chain scission reactions can also lead to an increase of $R_{i}$ which is attributed to cross-linking in the two-network theory.

### 3.5. Contribution of Physical Relaxation to Ageing 

Similar to stress relaxation, both physical and chemical ageing are observed in compression set (CS) measurements. According to the two-network theory [14], CS can be expressed as follows:

(10) CS=100·λ(Ri−Rc)+Rcλ(Ri−Rc)+Rc·λ213−100λ−1.

The samples that had aged in compression were released and then placed in a 100 °C oven for a day in order to recover towards physical equilibrium. This test procedure allows to separate the contribution of physical relaxation and chemical ageing since the latter cannot be reversed by heating [1]. 

**Figure 6:**
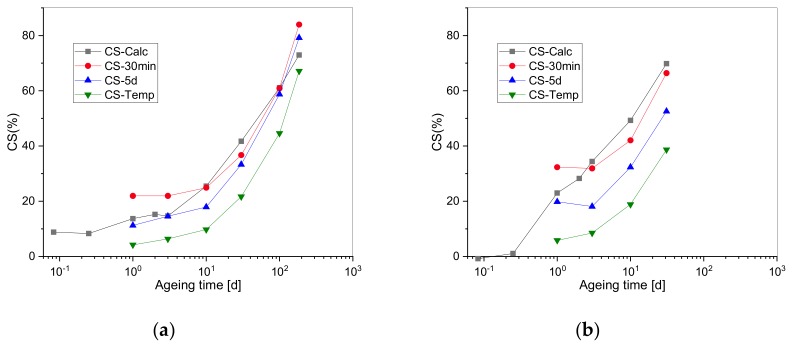
Compression set ($CS)\left(\%\right)$ after ageing at 125 °C versus ageing time for: (**a**) EPDM; (**b**) HNBR.

Figure 6 shows the compression set measurements of EPDM and HNBR as a function of ageing time after three different measurement times after release (30 min, 5 d, and after thermal conditioning at 100 °C) and the calculated CS with the two-network theory. CS values measured after 5 d were lower than those measured after 30 min and decreased further after tempering due to the recovery towards equilibrium. The difference between the equilibrium CS (after tempering) and the calculated CS might be due to the measurement of $R_{i}$ and $R_{c}$ that are determined in non-equilibrium under compression, while CS is measured after release from compression and in equilibrium (or very close to it).

When considering Equation (10), CS depends only on the ratio of chain scission to cross-linking and physical effects are not considered. In addition, the CS measurements of [14] were conducted 5 min after removal from the oven on 0.762 mm thin substance samples in tension. Another problem results from the fact that CS values are obtained at room temperature after cooling of the sample in compression, which may lead to the formation of stable secondary bonds that will contribute to fixing the deformed geometry [14] while $R_{c}$ measurements were carried out at the elevated ageing temperature. From Figure 6, one can observe the time dependency of CS values between release and measurement. Hence, to compare CS and $R_{c}$, one should consider a correction factor of CS data due to the thermal expansion at elevated temperature [38]. The coefficients of expansion of HNBR and EPDM are 1.25 × 10^−4^/K and 2 × 10^−4^/K, respectively, which correspond to correction factors of 0.964 and 0.944 for HNBR and EPDM, respectively, for a temperature difference of 102 °C. These correction factors are multiplied by the room-temperature compression set values to get the appropriate CS at 125 °C. 

**Figure 7:**
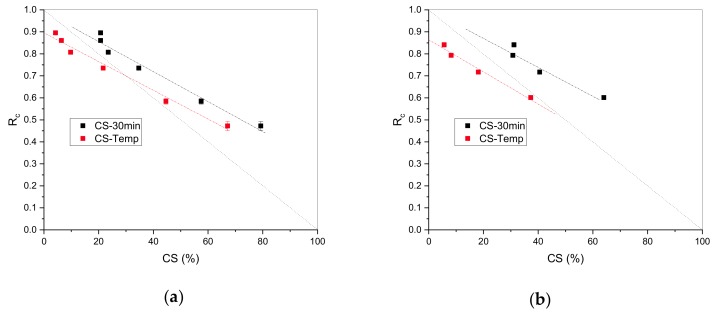
Continuous normalized stress relaxation ($R_{c})$ versus the corrected $CS\left(\%\right)$ after 30 min and $CS\left(\%\right)$ after tempering for: (**a**) EPDM; (**b**) HNBR.

The continuous normalized stress relaxation, $R_{c},$ versus the corrected CS after 30 min and CS after tempering are presented in Figure 7 (the dashed diagonal line is given as a guide for the eye for the perfect correlation between $R_{c}$ and CS). The evolution is linear, and the same slope is recorded for $R_{c}$ versus CS after 30 min and $R_{c}$ versus CS after tempering.

Since recovery is a physical phenomenon and chemical changes are irreversible, the contribution of physical to chemical processes are determined through the analysis of the corrected CS after tempering. Data of the corrected CS after tempering are taken as reference values and the intercept of the linear fit with $R_{c}$ corresponds to the shift factor to which the $R_{c}$ curve should be moved. This intercept represents the value at which irreversible chemical degradation begins. The difference between the shifted curve and the real one accounts for the physical processes that occur during chemical ageing. From Figure 7, the intercepts of the linear fits with $R_{c}$ are 0.893 and 0.862, respectively, for EPDM and HNBR. From these values, we can conclude that chemical processes become apparent below ~10% relaxation. The ageing times corresponding to $R_{c}$ values of 0.893 and 0.862 for EPDM and HNBR are used as the initiation times of the chemical degradation and are determined from the experimental $R_{c}$ curves (Figure 2). These are 1.1 d and 0.5 d for EPDM and HNBR, respectively. The corresponding CS values should be zero. The corrected CS values after tempering of 1 d aged EPDM and HNBR are 4.07% and 5.51%, which is very acceptable when considering the error measurements (standard deviation of $h_{2}$ from 10 measurements on an aged O-ring and of $h_{0}$ from 10 measurements on five unaged O-rings). Additionally, highly filled rubber compounds with about 80 phr of filler were used so that no ideal elastic recovery can be expected. 

## 4. Conclusions

It has been established on the basis of the experimental results that both chain scission and cross-linking occur during ageing of EPDM and HNBR. For HNBR cross-linking clearly dominates during longer ageing times, but for EPDM scission continues to compete cross-linking during extended ageing periods. A new approach to separate physical and chemical processes that occur during stress relaxation is proposed. The contribution of physical processes to the chemical ones, especially chain scission, during stress relaxation is determined by correlating equilibrium compression set data to continuous stress relaxation measurements. It has been shown that physical processes that happen during chemical ageing for longer ageing times should not be neglected, as their contribution to the total sealing force loss is over 10%. From the data analysis, characteristic times relative to the degradation process are also proposed. The physical relaxation time, the initiation time of chemical degradation, the induction time which corresponds to the threshold from which chain scission is noticeable, and the relaxation half-time are determined. These times are intrinsic only for the investigated rubbers (geometry and compounds) at the ageing temperature of 125 °C. For lower ageing temperatures longer times are to be expected.

The collected data and characteristic times will be of major importance for the development of a microphysical motivated model and for the identification of material parameters to predict the mechanical behaviour of rubber compounds that undergo microstructural changes, e.g., during ageing, that is in progress.

## Figures and Tables

**Table 1:** Relative deviation between axial and radial normalized average compressive force.

		Ageing Time [d]			
	3	10	30	100	185
**EPDM**	−1.2%	0.2%	3.5%	3.8%	5.3%
**HNBR**	5.5%	−3.4%	1.5%	--	--

EPDM—ethylene propylene diene rubber; HNBR—hydrogenated nitrile butadiene rubber.

**Table 2:** Characteristic times determined in continuous stress relaxation measurements (cf. Figure 2).

Material	Physical Relaxation (+) [d]	Relaxation Half-Time (*) [d]	Induction Time [d]
EPDM	0.06	169	10
HNBR	0.04	67	6
